# Supplementary material for: A Genome-Wide Functional Screen Identifies Enhancer and Protective Genes for Amyloid Beta-Peptide Toxicity
Source: Int J Mol Sci. 2023 Jan 9;24(2):1278. doi: 10.3390/ijms24021278 (PMC9865122; doi:10.3390/ijms24021278)
Supplement: Supplementary file 1 [file ijms-24-01278-s001.zip › ijms-2100939-supplementary.pdf]

SUPPLEMENTARY MATERIAL

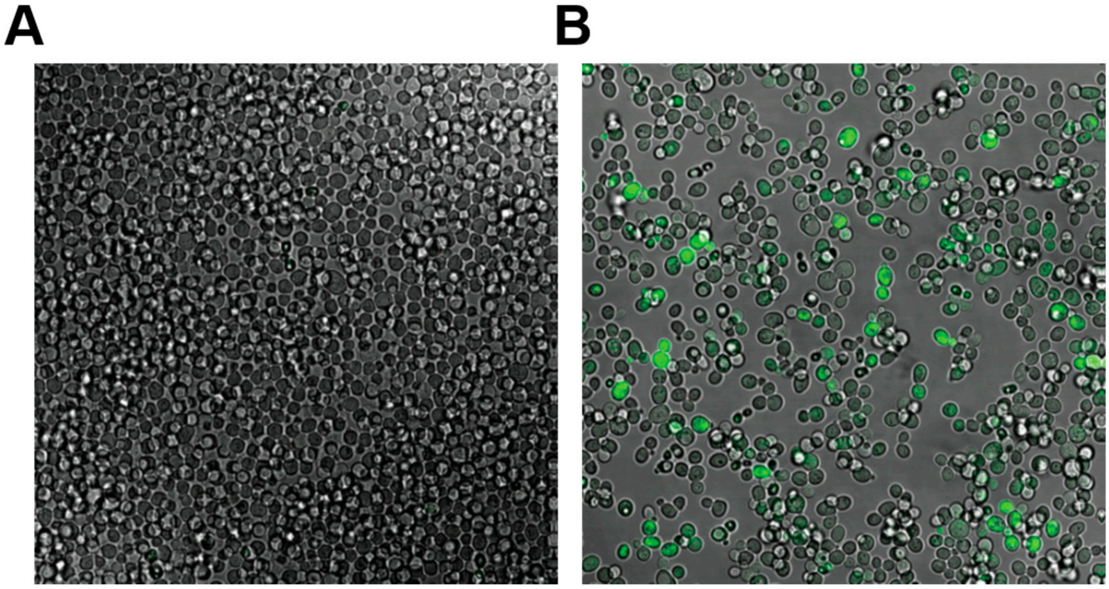

Figure S1. Induction of $A\beta_{1-42}$ expression in yeast. Representative confocal images of yeast transformed with the $A\beta_{1-42}$ construct and cultured for 6 h at 30°C in (**A**) non-inducing (glucose) and (**B**) inducing (galactose) medium.

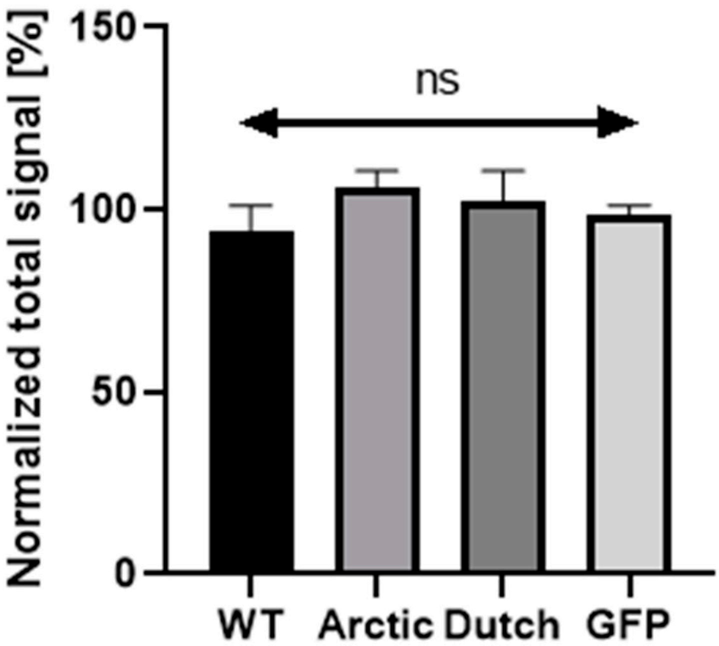

Figure S2. Western blots, as shown in Figure 2B, from different experiments were quantified to analyze the expression levels of Aβ_{1-42}-GFP using an anti-GFP antibody. All GFP bands were quantified for each condition and shown in the graph. Data are the mean ± SEM of 3-4 independent experiments. non-significant (ns) vs the respective conditions by ANOVA plus Bonferroni as post-hoc test.

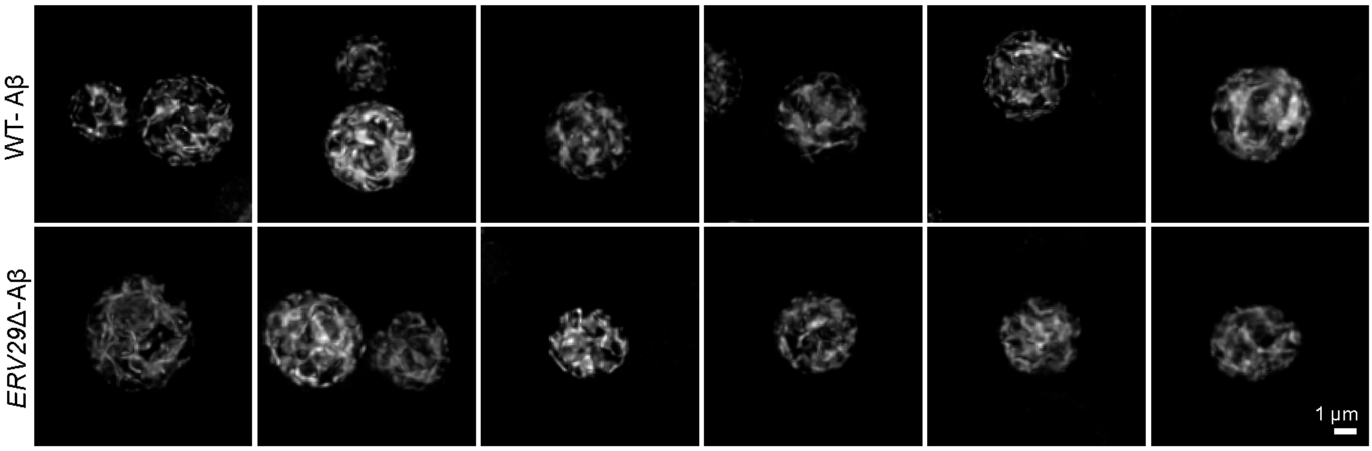

Figure S3. Projections of super-resolution microscopy images showing the subcellular localization of Aβ1-42-GFP in the WT and erv29D strains (yeasts lacking *SURF4*) transformed with the Aβ1-42-GFP construct and grown in inducing medium for 15 h at 30ºC.

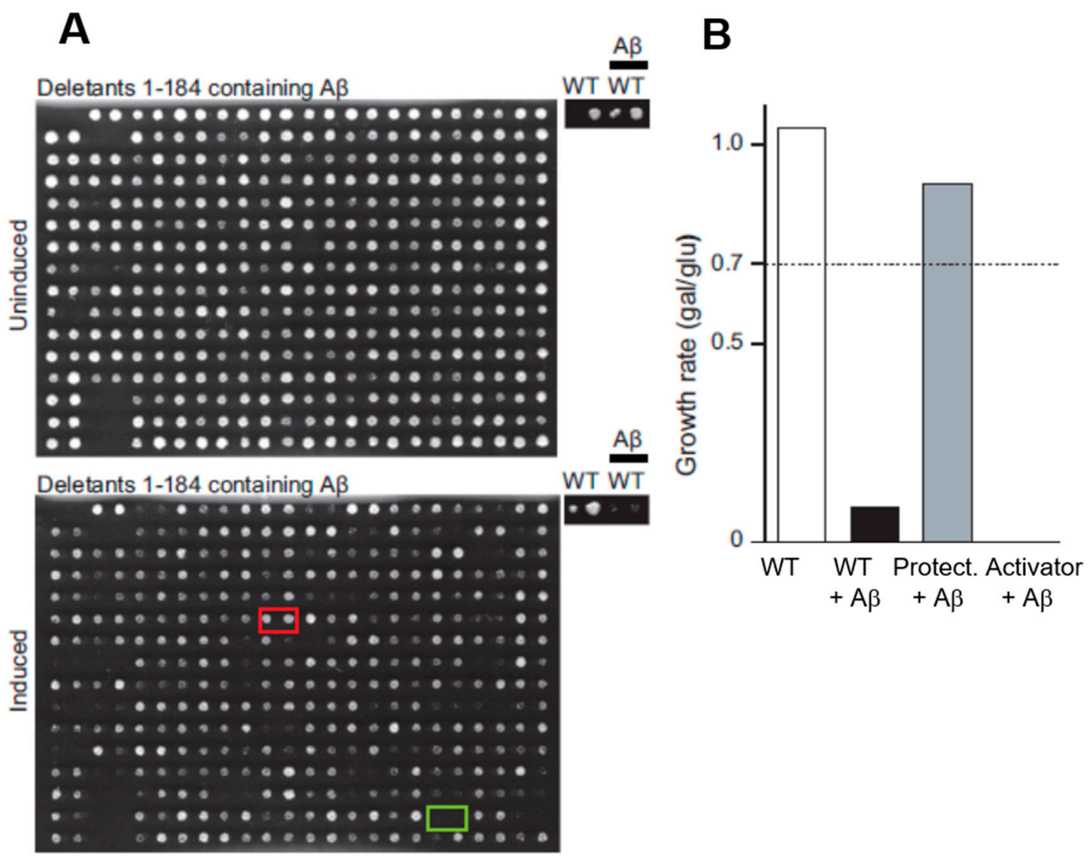

Figure S4. Screen plate for the first 184 knock-out strains assessed. (**A**) The strain containing $A\beta_{1\text{-}42}$ construct was mated with a library with of 5,154 gene knock-out strains. Resultant haploid cells were spotted twice on medium containing galactose (induced) or glucose (uninduced) and cultured for 3, 4 and 5 days at 30 °C. Image of the uninduced and the induced plate, at day 4, of the first 184 knock-outs containing the $A\beta_{1\text{-}42}$ construct (left) and the plate of the WT strain containing or not the $A\beta_{1\text{-}42}$ construct (right). An example of a knock-out that reverts $A\beta_{1\text{-}42}$ toxicity (red) and a knock-out that enhances $A\beta_{1\text{-}42}$ toxicity (green) is indicated. (**B**) Growth rate quantification of the WT strain containing or not $A\beta_{1\text{-}42}$ and an example of a revertant and an enhancer knock-out strain of $A\beta_{1\text{-}42}$ toxicity. The minimum growth rate set for the protective is at 0.7 whereas the maximum growth rate set for the enhancers is at 0, as indicated.

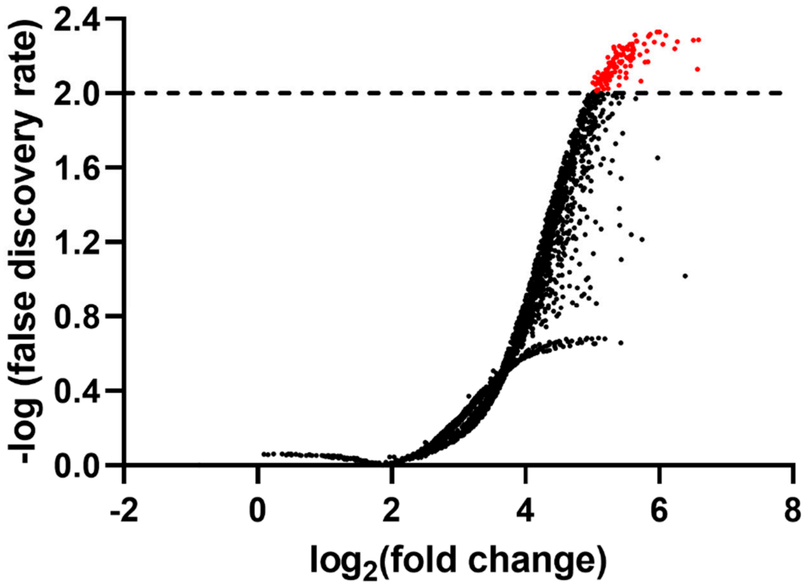

Figure S5. Identification of enhancers of Aβ1-42 toxicity by volcano plot. Data from day 3 was used for identification of the enhancers of Aβ1-42 toxicity. Statistical analysis was performed comparing each KO strain vs a non-KO using a non-parametric ANOVA with an Uncorrected Dunn's test posthoc test. In red are the strains with $p<0.001$.

Table S1. Enhancer genes of amyloid toxicity in yeast and their mammalian orthologues

Systematic name	Yeast gene	Function	Mammalian gene name
YAL011W	*SWC3*	SWr Complex, protein of unknown function	w/o orthologue
YAL021C	*CCR4*	Carbon Catabolite Repression, component of the CCR4-NOT transcriptional complex	*ANGEL1, LOC729530, CCRN4L,ANGEL2,2'-PDE*
YAL040C	*CLN3*	CycLiN, g1 cyclin involved in cell cycle progression	*CCNB2, CNTD2*
YAL044C	*GCV3*	GlyCine cleaVage, h subunit of the mitochondrial glycine decarboxylase complex	*LOC729080*
YAL060W	*BDH1*	Butanediol DeHydrogenase, nAD-dependent	*SORD*
YBL071C-B	unass.	Putative protein of unknown function	w/o orthologue
YBR048W	*RPS11B*	Ribosomal Protein of the Small subunit, protein component of the small (40S) ribosomal subunit	*RPS11*
YBR058C-A	*TSC3*	Temperature-sensitive Suppressors of Csg2 mutants, protein that stimulates the activity of serine palmitoyltransferase	w/o orthologue
YBR066C	*NRG2*	Negative Regulator of Glucose-controlled genes, transcriptional repressor	*ZBTB16,KLF14,KLF7 KLF5,*
YBR181C	*RPS6B*	Ribosomal Protein of the Small subunit, protein component of the small (40S) ribosomal subunit	*RPS6*
YBR186W	*PCH2*	Pachytene CHeckpoint, hexameric ring ATPase that remodels chromosome axis protein Hop1p	*TRIP13*
YBR206W	unass.	Dubious open reading frame	w/o orthologue
YBR214W	*SDS24*	Homolog of S. pombe SDS23, protein involved in cell separation during budding	w/o orthologue
YBR215W	*HPC2*	Histone Periodic Control, subunit of the HIR complex	w/o orthologue
YBR246W	*RRT2*	Regulator of rDNA Transcription, methylesterase performing penultimate step of diphthamide biosynthesis	*DPH7*
YBR266C	*SLM6*	Synthetic Lethal with Mss4, protein with a potential role in actin cytoskeleton organization	w/o orthologue
YBR267W	*REI1*	REquired for Isotropic bud growth, cytoplasmic pre-60S factor	*ZNF622*
YBR291C	*CTP1*	Citrate Transport Protein, mitochondrial inner membrane citrate transporter	*SLC25A1*
YCR020W-B	*HTL1*	High-Temperature Lethal, component of the RSC chromatin remodeling complex	w/o orthologue

YCR061W	*TVS1*	Protein of unknown function	w/o orthologue
YCR094W	*CDC50*	Cell Division Cycle, endosomal protein that interacts with phospholipid flippase Drs2p	*TMEM30A, TMEM30B*
YDL009C	unass.	Protein of unknown function	w/o orthologue
YDL063C	*SYO1*	SYnchronized impOrt or SYmpOrtin, transport adaptor or symportin	*HEATR3*
YDL075W	*RPL31A*	Ribosomal Protein of the Large subunit, ribosomal 60S subunit protein L31A	*LOC641311*
YDL080C	*THI3*	THIamine metabolism, regulatory protein that binds Pdc2p and Thi2p transcription factors	w/o orthologue
YDL101C	*DUN1*	DNA-damage UNinducible, cell-cycle checkpoint serine-threonine kinase	*CHEK2*
YDR123C	*INO2*	INOsitol requiring, transcription factor	w/o orthologue
YDR153C	*ENT5*	Epsin N-Terminal homology, protein containing an N-terminal epsin-like domain	*EPN1*
YDR159W	*SAC3*	Suppressor of ACtin, mRNA export factor	*MCM3AP, SAC3D1*
YDR171W	*HSP42*	Heat Shock Protein, small heat shock protein (sHSP) with chaperone activity	w/o orthologue
YDR174W	*HMO1*	High MObility group (HMG) family, chromatin associated high mobility group (HMG) family member	*HMG20A*
YDR186C	*SND1*	Putative protein of unknown function	w/o orthologue
YDR195W	*REF2*	RNA End Formation, rNA-binding protein	w/o orthologue
YDR214W	*AHA1*	Activator of Heat shock protein 90 ATPase, co-chaperone that binds Hsp82p and activates its ATPase activity	*AHSA1*
YDR219C	*MFB1*	Mitochondria-associated F-Box protein, mitochondria-associated F-box protein	w/o orthologue
YDR225W	*HTA1*	Histone h Two A, histone H2A	*H2AFB2*
YDR378C	*LSM6*	Like SM, lsm (Like Sm) protein	*LSM6, SNRPF*
YDR537C	unass.	Dubious open reading frame	w/o orthologue
YER016W	*BIM1*	BInding to Microtubules, microtubule plus end-tracking protein	*MAPRE1, MAPRE2, MAPRE3*
YER066W	*RRT13*	Regulator of rDNA Transcription, putative protein of unknown function	*FBXW7*
YER068W	*MOT2*	Modulator Of Transcription, ubiquitin-protein ligase subunit of the CCR4-NOT complex	*CNOT4*
YER092W	*IES5*	Ino Eighty Subunit, non-essential INO80 chromatin remodeling complex subunit	w/o orthologue
YER119C-A	unass.	Dubious open reading frame	w/o orthologue

YER151C	*UBP3*	UBiquitin-specific Protease, ubiquitin-specific protease involved in transport and osmotic response	*USP10*
YFR034C	*PHO4*	PHOsphate metabolism, basic helix-loop-helix (bHLH) transcription factor of the myc-family	*MLXIP*
YFR036W	*CDC26*	Cell Division Cycle, subunit of the Anaphase-Promoting Complex/Cyclosome (APC/C)	w/o orthologue
YGL007C-A	unass.	Putative protein of unknown function	w/o orthologue
YGL041C-B	unass.	Putative protein of unknown function	w/o orthologue
YGL168W	*HUR1*	HydroxyUrea Resistance, protein of unknown function	w/o orthologue
YGL222C	*EDC1*	Enhancer of mRNA DeCapping, rNA-binding protein that activates mRNA decapping directly	w/o orthologue
YGL241W	*KAP114*	KAryoPherin, karyopherin	*IPO9*
YGL253W	*HXK2*	HeXoKinase, hexokinase isoenzyme 2	*GCK, HKDC1, HK2*
YGR006W	*PRP18*	Pre-mRNA Processing, splicing factor and component of snRNP U5	*PRPF18*
YGR015C	*EAT1*	Putative protein of unknown function	*ABHD11*
YGR081C	*SLX9*	Protein required for pre-rRNA processing	w/o orthologue
YGR133W	*PEX4*	PEroXin, peroxisomal ubiquitin conjugating enzyme	*UBE2F*
YGR226C	unass.	Dubious open reading frame	w/o orthologue
YGR271C-A	*EFG1*	Exit From G1, essential protein required for maturation of 18S rRNA	w/o orthologue
YGR284C	*ERV29*	ER Vesicle, protein localized to COPII-coated vesicles	*SURF4*
YHR021C	*RPS27B*	Ribosomal Protein of the Small subunit, protein component of the small (40S) ribosomal subunit	*RPS27*
YHR025W	*THR1*	THReonine requiring, homoserine kinase	w/o orthologue
YHR064C	*SSZ1*	Hsp70 protein that interacts with Zuo1p (a DnaJ homolog)	*HSPA2,HSPA8,HSPA14, HSPA6,*
YIL154C	*IMP2*	Independent of Mitochondrial Particle, transcriptional activator involved in maintenance of ion homeostasis	w/o orthologue
YIL156W	*UBP7*	UBiquitin-specific Protease, ubiquitin-specific protease that cleaves ubiquitin-protein fusions	*USP28,USP29,USP37,USP50, USP8,USP32,USP51,USP26, USP2,USP21,*
YIL157C	*COA1*	Cytochrome Oxidase Assembly, mitochondrial inner membrane protein	w/o orthologue
YJL012C-A	unass.	Merged open reading frame	w/o orthologue
YJL027C	unass.	Putative protein of unknown function	w/o orthologue

YJL080C	*SCP160*	S. cerevisiae protein involved in the Control of Ploidy, essential RNA-binding G protein effector of mating response pathway	*HDLBP*
YJL123C	*MTC1*	Maintenance of Telomere Capping, protein of unknown function that may interact with ribosomes	w/o orthologue
YJL124C	*LSM1*	Like SM, lsm (Like Sm) protein	*LSM1*
YJL141C	*YAK1*	Yet Another Kinase, serine-threonine protein kinase	*DYRK1A/B,HIPK3,HIPK4, HIPK1,*
YJL176C	*SWI3*	SWItching deficient, subunit of the SWI/SNF chromatin remodeling complex	*SMARCC2, SMARCC1*
YJL188C	*BUD19*	BUD site selection, dubious open reading frame	w/o orthologue
YKL009W	*MRT4*	mRNA Turnover 4, protein involved in mRNA turnover and ribosome assembly	*LOC124865*
YKL096C-B	unass.	Putative protein of unknown function	w/o orthologue
YKL139W	*CTK1*	Carboxy-Terminal domain Kinase, catalytic (alpha) subunit of C-terminal domain kinase I (CTDK-I)	*CDC2L5, CDK9*
YKL163W	*PIR3*	Protein containing Internal Repeats, o-glycosylated covalently-bound cell wall protein	w/o orthologue
YKR024C	*DBP7*	Dead Box Protein, putative ATP-dependent RNA helicase of the DEAD-box family	*DDX31*
YKR095W	*MLP1*	Myosin-Like Protein, myosin-like protein associated with the nuclear envelope	*TPR,LEKR1,GOLGA4,CENPE, CENPF,GOLGB1,*
YLL002W	*RTT109*	Regulator of Ty1 Transposition, histone acetyltransferase	w/o orthologue
YLL013C	*PUF3*	PUmilio-homology domain Family, protein of the mitochondrial outer surface	*PUM2, PUM1*
YLL024C	*SSA2*	Stress-Seventy subfamily A, aTP-binding protein	*HSPA8,HSPA1B,HSPA6,HSPA2, HSPA1A,HSPA1L,HSPA5,*
YLL043W	*FPS1*	Fdp1 Suppressor, aquaglyceroporin	*AQP3 to 5, AQP7, AQP 7, AQP 9 &10, LOC100509620*
YLL046C	*RNP1*	Ribonucleoprotein that contains two RNA recognition motifs (RRM)	w/o orthologue
YLR015W	*BRE2*	BREfeldin A sensitivity, subunit of COMPASS (Set1C) complex	*ASH2L*
YLR046C	unass.	Putative membrane protein	w/o orthologue
YLR047C	*FRE8*	Protein with sequence similarity to iron/copper reductases	w/o orthologue
YLR048W	*RPS0B*	Ribosomal Protein of the Small subunit, protein component of the small (40S) ribosomal subunit	*RPSAP15*
YLR057W	*MNL2*	MaNnosidase-Like protein, putative mannosidase involved in ER-associated protein degradation	*MAN1B1,MAN1C1,MAN1A2, MAN1A1,*

YLR061W	*RPL22A*	Ribosomal Protein of the Large subunit, ribosomal 60S subunit protein L22A	*RPL22, RPL22L1*
YLR062C	*BUD28*	BUD site selection, dubious open reading frame	w/o orthologue
YLR063W	*BMT6*	Base Methyltransferase of Twenty five S rRNA 6, methyltransferase required for m3U2843 methylation of the 25S rRNA	w/o orthologue
YLR072W	*LAM6*	Lipid transfer protein Anchored at Membrane contact site, protein of unknown function	*GRAMD1A,GRAMD1B, GRAMD1C, GRAMD2A*
YLR079W	*SIC1*	Substrate/Subunit Inhibitor of Cyclin-dependent protein kinase, cyclin-dependent kinase inhibitor (CKI)	w/o orthologue
YLR182W	*SWI6*	SWItching deficient, transcription cofactor	w/o orthologue
YLR322W	*VPS65*	Vacuolar Protein Sorting, dubious open reading frame	w/o orthologue
YLR369W	*SSQ1*	Stress-Seventy subfamily Q, mitochondrial hsp70-type molecular chaperone	*HSPA9*
YLR418C	*CDC73*	Cell Division Cycle, component of the Paf1p complex	*CDC73*
YLR433C	*CNA1*	CalciNeurin A, calcineurin A	*PPP3CA,B,C*
YML009W-B	unass.	Dubious open reading frame	w/o orthologue
YML012C-A	unass.	Dubious open reading frame	w/o orthologue
YML014W	*TRM9*	TRna Methyltransferase, tRNA methyltransferase	*ALKBH8, TRMT9B*
YML024W	*RPS17A*	Ribosomal Protein of the Small subunit, ribosomal protein 51 (rp51) of the small (40s) subunit	*RPS17*
YML050W	*AIM32*	Altered Inheritance rate of Mitochondria, putative protein of unknown function	w/o orthologue
YML061C	*PIF1*	Petite Integration Frequency, dNA helicase	*PIF1*
YMR018W	*PEX9*	Putative protein of unknown function with similarity to human PEX5Rp	*PEX5*
YMR116C	*ASC1*	Absence of growth Suppressor of Cyp1, g-protein beta subunit and guanine dissociation inhibitor for Gpa2p	*RACK1*
YMR179W	*SPT21*	SuPpressor of Ty, protein with a role in transcriptional silencing	w/o orthologue
YMR198W	*CIK1*	Chromosome Instability and Karyogamy, kinesin-associated protein	w/o orthologue
YMR222C	*FSH2*	Family of Serine Hydrolases, putative serine hydrolase that localizes to the cytoplasm	*OVCA2, DHFRP1*
YMR242W-A	unass.	Putative protein of unknown function	w/o orthologue
YNL022C	*RCM1*	rRNA m5C methyltransferase	*NSUN7*
YNL027W	*CRZ1*	Calcineurin-Responsive Zinc finger, transcription factor	*SP3, SP9, KLF10, SP5, SP1, OSR1, KLF17, KLF11, SP6*

YNL076W	*MKS1*	Multicopy Kinase Suppressor, pleiotropic negative transcriptional regulator	w/o orthologue
YNL138W	*SRV2*	Suppressor of RasVal19, cAP (cyclase-associated protein)	*CAP2, CAP1*
YNL146W	unass.	Putative protein of unknown function	w/o orthologue
YNL220W	*ADE12*	adenylsuccinate synthesis; ADEnine requiring, catalyzes the first step in synthesis of adenosine monophosphate	*ADSS1*
YNR052C	*POP2*	PGK promoter directed OverProduction, rNase of the DEDD superfamily	*CNOT8,CNOT7,*
YOL045W	*PSK2*	Pas domain-containing Serine/threonine protein Kinase, pAS-domain containing serine/threonine protein kinase	*PIM2,PIM3,*
YOL046C	unass.	Dubious open reading frame	w/o orthologue
YOL054W	*PSH1*	Pob3/Spt16 Histone associated, e3 ubiquitin ligase targeting centromere-binding protein Cse4p	*TRIM8,TRIM3,TRIM2, TRIM25,TRIM40,*
YOL085C	unass.	Dubious open reading frame	w/o orthologue
YOR113W	*AZF1*	Asparagine-rich Zinc-Finger, zinc-finger transcription factor	*SP1, FEZF2, FEZP1*
YOR120W	*GCY1*	Galactose-inducible Crystallin-like Yeast protein, glycerol dehydrogenase	*AKR1B10,AKR1B1,AKR1A1, AKR1D1,AKR1C2,AKR1C1, AKR1E2,AKR1C3,AKR1C4, AKR1B15,*
YOR141C	*ARP8*	Actin-Related Protein, nuclear actin-related protein involved in chromatin remodeling	*ACTR8*
YOR196C	*LIP5*	LIPoic acid, protein involved in biosynthesis of the coenzyme lipoic acid	*LIAS*
YOR301W	*RAX1*	Protein involved in bud site selection during bipolar budding	*AKAP10*
YOR309C	unass.	Dubious open reading frame	w/o orthologue
YOR312C	*RPL20B*	Ribosomal Protein of the Large subunit, ribosomal 60S subunit protein L20B	*RPL18A*
YPL008W	*CHL1*	CHromosome Loss, probable DNA helicase	*TNFRSF6B, BRIP1*
YPL098C	*MGR2*	Mitochondrial Genome Required, subunit of the TIM23 translocase complex	*ROMO1*
YPL106C	*SSE1*	ATPase component of heat shock protein Hsp90 chaperone complex	*HYOU1,HSPA4L,HSPA4,*
YPL145C	*KES1*	KrE11-1 Suppressor, one of seven members of the yeast oxysterol binding protein family	*OSBPL9, OSBPL11, OSBPL10, OSBPL8, OSBPL5*
YPL174C	*NIP100*	Nuclear ImPort, large subunit of the dynactin complex	*CLIP3,CLIP2,CLIP4,CLIP1, CEP350,*
YPL178W	*CBC2*	Cap Binding Complex, small subunit of the heterodimeric cap binding complex with Sto1p	*NCBP2*

YPL240C	*HSP82*	Heat Shock Protein, hsp90 chaperone	*HSP90AA1*
YPR043W	*RPL43A*	Ribosomal Protein of the Large subunit, ribosomal 60S subunit protein L43A	*RPL37A*
YPR057W	*BRR1*	Bad Response to Refrigeration, snRNP protein component of spliceosomal snRNPs	w/o orthologue
YPR096C	unass.	Protein of unknown function	w/o orthologue
YPR159C-A	unass.	Protein of unknown function	w/o orthologue
YPR159W	*KRE6*	Killer toxin REsistant, type II integral membrane protein	w/o orthologue

unass. = unassigned
w/o orthologue = without mammalian orthologue

Table S2. Protective genes regarding amyloid toxicity in yeast and their mammalian orthologues

Systematic Name	Yeast Gene	Function	Mammalian Gene Name
YAL039C	CYC3	CYtochrome C heme lyase (holocytochrome c synthase)	HCCS
YBL007C	SLA1	Synthetic Lethal with ABP1, cytoskeletal protein binding protein	w/o orthologue
YBL022C	PIM1	Proteolysis In Mitochondria, ATP-dependent Lon protease	LONP1
YBL024W	NCL1	NuCLear protein, s-adenosyl-L-methionine-dependent tRNA: m5C-methyltransferase	NSUN2
YBL038W	MRPL16	Mitochondrial Ribosomal Protein, Large subunit	MRPL16
YBL045C	COR1	CORe protein of QH2 cytochrome c reductase	UQCRC1
YBL051C	PIN4	Psi+ INducibility, involved in G2/M phase progression and response to DNA damage	w/o orthologue
YBL052C	SAS3	Something About Silencing, histone acetyltransferase catalytic subunit of NuA3 complex	w/o orthologue
YBL080C	PET112	PETite colonies, subunit of the trimeric GatFAB AmidoTransferase(AdT) complex	GATB
YBL090W	MRP21	Mitochondrial Ribosomal Protein of the small subunit	w/o orthologue
YBL098W	BNA4	Biosynthesis of Nicotinic Acid, kynurenine 3-monooxygenase	KMO
YBL106C	SRO77	Suppressor of rho3, protein with roles in exocytosis and cation homeostasis	LLGL1
YBR021W	FUR4	5-FlUoRouridine sensitivity, plasma membrane localized uracil permease	w/o orthologue
YBR107C	IML3	Increased Minichromosome Loss, outer kinetochore protein and component of the Ctf19 complex	w/o orthologue
YBR113W	unass.	Dubious open reading frame	w/o orthologue
YBR114W	RAD16	RADiation sensitive, nucleotide excision repair (NER) protein	ZRANB3
YBR120C	CBP6	Cytochrome B Protein synthesis, mitochondrial protein required for translation of the COB mRNA	w/o orthologue
YBR131W	CCZ1	Calcium Caffeine Zinc sensitivity, subunit of a heterodimeric guanine nucleotide exchange factor (GEF)	w/o orthologue
YBR163W	EXO5	EXOnuclease V, mitochondrial 5'-3' exonuclease and sliding exonuclease	EXO5
YBR241C	VVS1	Putative transporter	w/o orthologue

YBR280C	SAF1	SCF Associated Factor, f-Box protein involved in proteasome-dependent degradation of Aah1p	w/o orthologue
YBR282W	MRPL27	Mitochondrial Ribosomal Protein, large subunit	MRPL41
YBR283C	SSH1	Sec Sixty-one Homolog, subunit of the Ssh1 translocon complex	SEC61A1, SEC61A2
YBR288C	APM3	Clathrin Adaptor Protein complex Medium chain, mu3-like subunit of the clathrin associated protein complex (AP-3)	AP3M1, AP3M2
YBR296C	PHO89	PHOsphate metabolism, plasma membrane Na+/Pi cotransporter	w/o orthologue
YCR003W	MRPL32	Mitochondrial Ribosomal Protein, large subunit	MRPL32
YCR028C-A	RIM1	Replication In Mitochondria, ssDNA-binding protein essential for mitochondrial genome maintenance	SSBP1
YCR046C	IMG1	Integrity of Mitochondrial Genome, mitochondrial ribosomal protein of the large subunit	MRPL19
YCR053W	THR4	THReonine requiring, threonine synthase	THNSL1
YCR071C	IMG2	Integrity of Mitochondrial Genome, mitochondrial ribosomal protein of the large subunit	MRPL49
YCR073W-A	SOL2	Suppressor Of Los1-1, protein with a possible role in tRNA export	PGLS
YDL044C	MTF2	Mitochondrial Transcription Factor, mitochondrial protein that interacts with mitochondrial RNA polymerase	w/o orthologue
YDL067C	COX9	Cytochrome c OXidase, subunit VIIa of cytochrome c oxidase (Complex IV)	w/o orthologue
YDL069C	CBS1	Cytochrome B Synthesis, mitochondrial translational activator of the COB mRNA	w/o orthologue
YDL104C	QRI7	Protein involved in threonylcarbamoyl adenosine biosynthesis	OSGEPL1
YDL174C	DLD1	D-Lactate Dehydrogenase, d-lactate dehydrogenase	LDHD
YDL190C	UFD2	Ubiquitin Fusion Degradation, ubiquitin chain assembly factor (E4)	UBE4A
YDR028C	REG1	REsistance to Glucose repression, regulatory subunit of type 1 protein phosphatase Glc7p	w/o orthologue
YDR029W	unass.	Dubious open reading frame	w/o orthologue
YDR035W	ARO3	AROmatic amino acid requiring, 3-deoxy-D-arabino-heptulosonate-7-phosphate (DAHP) synthase	w/o orthologue

YDR069C	DOA4	Degradation Of Alpha, ubiquitin hydrolase	USP21, USP50, USP8
YDR077W	SED1	Suppression of Exponential Defect, major stress-induced structural GPI-cell wall glycoprotein	w/o orthologue
YDR079W	PET100	PETite colonies, chaperone that facilitates the assembly of cytochrome c oxidase	w/o orthologue
YDR114C	unass.	Putative protein of unknown function	w/o orthologue
YDR115W	MRX14	Mitochondrial organization of gene expression, putative mitochondrial ribosomal protein of the large subunit	MRPL34
YDR128W	MTC5	Maintenance of Telomere Capping, subunit of the SEA (Seh1-associated) complex	WDR59
YDR194C	MSS116	Mitochondrial Splicing System, mitochondrial transcription elongation factor	w/o orthologue
YDR197W	CBS2	Cytochrome B Synthesis, mitochondrial translational activator of the COB mRNA	w/o orthologue
YDR230W	unass.	Dubious open reading frame	w/o orthologue
YDR256C	CTA1	CaTalase A, catalase A	CAT
YDR268W	MSW1	Mitochondrial aminoacyl-tRNA Synthetase, tryptophan (W)	WARS2
YDR296W	MHR1	Mitochondrial Homologous Recombination, protein involved in homologous recombination in mitochondria	w/o orthologue
YDR298C	ATP5	ATP synthase, subunit 5 of the stator stalk of mitochondrial F1F0 ATP synthase	ATP5PO
YDR347W	MRP1	Mitochondrial Ribosomal Protein, mitochondrial ribosomal protein of the small subunit	w/o orthologue
YDR375C	BCS1	Ubiquinol-cytochrome c reductase (bc1) Synthesis, protein translocase and chaperone required for Complex III assembly	BCS1L
YDR377W	ATP17	ATP synthase, subunit f of the F0 sector of mitochondrial F1F0 ATP synthase	w/o orthologue
YDR405W	MRP20	Mitochondrial Ribosomal Protein, mitochondrial ribosomal protein of the large subunit	MRPL23
YDR421W	ARO80	AROmatic amino acid requiring, zinc finger transcriptional activator of the Zn2Cys6 family	w/o orthologue
YDR442W	unass.	Dubious open reading frame	w/o orthologue
YDR443C	SSN2	Suppressor of SNf1, subunit of the RNA polymerase II mediator complex	SLC50A1

YDR529C	QCR7	UbiQuinol-cytochrome C oxidoReductase, subunit 7 of ubiquinol cytochrome-c reductase (Complex III)	UQCRB
YEL071W	DLD3	D-Lactate Dehydrogenase, d-lactate dehydrogenase	D2HGDH
YER005W	YND1	Yeast Nucleoside Diphosphatase, apyrase with wide substrate specificity	ENTPD4, ENTPD7
YER017C	AFG3	ATPase Family Gene, mitochondrial inner membrane m-AAA protease component	AFG3L2
YER035W	EDC2	Enhancer of mRNA DeCapping, rNA-binding protein that directly activates mRNA decapping	w/o orthologue
YER042W	MXR1	Peptide Methionine sulfoXide Reductase, methionine-S-sulfoxide reductase	MSRA
YER050C	RSM18	Ribosomal Small subunit of Mitochondria, mitochondrial ribosomal protein of the small subunit	MRPS18C
YER055C	HIS1	HIStidine, aTP phosphoribosyltransferase	w/o orthologue
YER058W	PET117	PETite colonies, protein required for assembly of cytochrome c oxidase	PET117
YER060W-A	FCY22	FluoroCYtosine resistance, putative purine-cytosine permease	w/o orthologue
YER084W	unass.	Protein of unknown function	w/o orthologue
YER087W	AIM10	Altered Inheritance rate of Mitochondria, protein with similarity to tRNA synthetases	PARS2
YER108C	unass.	Merged ORF	w/o orthologue
YER134C	unass.	Magnesium-dependent acid phosphatase	w/o orthologue
YER153C	PET122	PETite colonies, mitochondrial translational activator specific for the COX3 mRNA	w/o orthologue
YER154W	OXA1	Cytochrome OXidase Activity, mitochondrial inner membrane insertase	OXA2
YFL030W	AGX1	Alanine:Glyoxylate aminotrans(X)ferase, alanine:glyoxylate aminotransferase (AGT)	AGXT
YFL036W	RPO41	RNA POlymerase, mitochondrial RNA polymerase	POLRMT
YFR024C	LSB3	Deleted ORF	w/o orthologue
YGL003C	CDH1	CDC20 Homolog, activator of anaphase-promoting complex/cyclosome (APC/C)	FZR1
YGL034C	unass.	Dubious open reading frame	w/o orthologue
YGL042C	unass.	Dubious open reading frame	w/o orthologue

YGL064C	MRH4	Mitochondrial RNA Helicase, mitochondrial ATP-dependent RNA helicase of the DEAD-box family	DDX28
YGL107C	RMD9	Required for Meiotic nuclear Division, mitochondrial protein required for respiratory growth	w/o orthologue
YGL119W	COQ8	COenzyme Q, protein required for ubiquinone biosynthesis and respiratory growth	COQ8A, COQ8B
YGL124C	MON1	MONensin sensitivity, subunit of a heterodimeric guanine nucleotide exchange factor (GEF)	MON1A, MON1B
YGL125W	MET13	METhionine requiring, major isozyme of methylenetetrahydrofolate reductase	MTHFR
YGL126W	SCS3	Suppressor of Choline Sensitivity, protein required for inositol prototrophy	FITM2
YGL129C	RSM23	Ribosomal Small subunit of Mitochondria, mitochondrial ribosomal protein of the small subunit	DAP3
YGL143C	MRF1	Mitochondrial peptide chain Release Factor, mitochondrial translation release factor	MTRF1, MTRF1L
YGL148W	ARO2	AROmatic amino acid requiring, bifunctional chorismate synthase and flavin reductase	w/o orthologue
YGL186C	TPN1	Transport of PyridoxiNe, plasma membrane pyridoxine (vitamin B6) transporter	w/o orthologue
YGL205W	POX1	Fatty-acyl coenzyme A oxidase	ACOXL
YGL208W	SIP2	SNF1-Interacting Protein, one of three beta subunits of the Snf1 kinase complex	PRKAB1, PRKAB2
YGL215W	CLG1	Cyclin-Like Gene, cyclin-like protein that interacts with Pho85p	CNPPD1
YGL255W	ZRT1	Zinc-Regulated Transporter, high-affinity zinc transporter of the plasma membrane	SLC39A1
YGR034W	RPL26B	Ribosomal Protein of the Large subunit, ribosomal 60S subunit protein L26B	RPL26
YGR035C	unass.	Putative protein of unknown function	w/o orthologue
YGR043C	NQM1	Non-Quiescent Mutant, transaldolase of unknown function	TALDO1
YGR062C	COX18	Cytochrome c OXidase, protein required for membrane insertion of C-terminus of Cox2p	COX18
YGR070W	ROM1	RhO1 Multicopy suppressor, gDP/GTP exchange protein (GEP) for Rho1p	ARHGEF3, NET1

YGR093W	DRN1	Debranching enzyme-associated RiboNuclease, splicing factor that modulates turnover of branched RNAs by Dbr1p	CWF19L1
YGR102C	GTF1	Glutaminyl Transamidase subunit F, subunit of the trimeric GatFAB AmidoTransferase(AdT) complex	w/o orthologue
YGR112W	SHY1	SURF Homolog of Yeast, mitochondrial inner membrane protein required for complex IV assembly	SURF1
YGR129W	SYF2	SYnthetic lethal with cdc40 (Forty), member of the NineTeen Complex (NTC)	w/o orthologue
YGR138C	TPO2	Transporter of POlyamines, polyamine transporter of the major facilitator superfamily	w/o orthologue
YGR150C	CCM1	COB and COX1 mRNA maturation, mitochondrial 15S rRNA-binding protein	w/o orthologue
YGR165W	MRPS35	Mitochondrial Ribosomal Protein, Small subunit	w/o orthologue
YGR171C	MSM1	Mitochondrial aminoacyl-tRNA Synthetase, Methionine	MARS2
YGR204W	ADE3	ADEnine requiring, cytoplasmic trifunctional enzyme C1-tetrahydrofolate synthase	MTHFD1
YGR215W	RSM27	Ribosomal Small subunit of Mitochondria, mitochondrial ribosomal protein of the small subunit	w/o orthologue
YGR220C	MRPL9	Mitochondrial Ribosomal Protein, Large subunit	MRPL3
YGR222W	PET54	PETite colonies, mitochondrial inner membrane protein	w/o orthologue
YGR255C	COQ6	COenzyme Q, putative flavin-dependent monooxygenase	COQ6
YGR257C	MTM1	Manganese Trafficking factor for Mitochondrial SOD2, mitochondrial protein of the mitochondrial carrier family	w/o orthologue
YHL006C	SHU1	Suppressor of HU sensitivity, component of Shu complex (aka PCSS complex)	w/o orthologue
YHL008C	unass.	Putative protein of unknown function	w/o orthologue
YHL010C	ETP1	Ethanol Tolerance Protein, putative protein of unknown function required for growth on ethanol	BRAP
YHL019C	APM2	Clathrin Adaptor Protein complex Medium chain, protein of unknown function	w/o orthologue
YHL034C	SBP1	Protein that binds eIF4G and has a role in repression of translation	w/o orthologue

YHL035C	VMR1	Vacuolar Multidrug Resistance, vacuolar membrane protein	CFTR
YHL038C	CBP2	Cytochrome B mRNA Processing, required for splicing of the group I intron bI5 of the COB pre-mRNA	w/o orthologue
YHL045W	PXP3	Putative protein of unknown function	w/o orthologue
YHL046C	PAU13	SeriPAUperin, protein of unknown function	w/o orthologue
YHL047C	ARN2	AFT1 ReguloN, transporter	w/o orthologue
YHR011W	DIA4	Digs Into Agar, probable mitochondrial seryl-tRNA synthetase	SARS2
YHR012W	VPS29	Vacuolar Protein Sorting, subunit of the membrane-associated retromer complex	VPS29
YHR015W	MIP6	Mex67-Interacting Protein, putative RNA-binding protein	w/o orthologue
YHR037W	PUT2	Proline UTilization, delta-1-pyrroline-5-carboxylate dehydrogenase	ALDH4A1
YHR050W-A		Protein of unknown function	w/o orthologue
YHR051W	COX6	Cytochrome c OXidase, subunit VI of cytochrome c oxidase (Complex IV)	COX5A
YHR116W	COX23	Cytochrome OXidase, protein that functions in mitochondrial copper homeostasis	CHCHD7
YHR147C	MRPL6	Mitochondrial Ribosomal Protein, Large subunit	RPL9
YHR151C	MTC6	Maintenance of Telomere Capping, protein of unknown function	w/o orthologue
YHR155W	YSP1	Yeast Suicide Protein, mitochondrial protein	CCNY
YHR168W	MTG2	MiTochondrial Gtpase 2, putative GTPase	GTPBP10, MTG2
YHR198C	AIM18	Altered Inheritance rate of Mitochondria, putative protein of unknown function	w/o orthologue
YIL002C	INP51	INositol polyphosphate 5-Phosphatase, phosphatidylinositol 4	SYNJ1, SYNJ2
YIL006W	YIA6	Mitochondrial NAD+ transporter	SLC25A32
YIL036W	CST6	Chromosome STability, basic leucine zipper (bZIP) transcription factor	ATF2, CREB5
YIL039W	TED1	Trafficking of Emp24p/Erv25p-dependent cargo Disrupted, gPI-glycan remodelase	MPPE1
YIL041W	GVP36	Golgi Vesicle Protein, bAR domain protein that localizes to early and late Golgi vesicles	w/o orthologue
YIL049W	DFG10	Defective for Filamentous Growth, probable polyprenol reductase	SRD5A3

YIL050W	PCL7	Pho85 CycLin, pho85p cyclin of the Pho80p subfamily	w/o orthologue
YIL053W	GPP1	Glycerol-3-Phosphate Phosphatase, constitutively expressed DL-glycerol-3-phosphate phosphatase	w/o orthologue
YIL064W	EFM4	Elongation Factor Methyltransferase, lysine methyltransferase	EEF1AKMT2
YIL071C	PCI8	Proteasome-COP9 signalosome (CSN)-eIF3, possible shared subunit of Cop9 signalosome (CSN) and eIF3	GPS1
YIL073C	SPO22	SPOrulation, meiosis-specific protein essential for chromosome synapsis	TEX11
YIL077C		Putative protein of unknown function	w/o orthologue
YIL092W		Putative protein of unknown function	w/o orthologue
YIL114C	POR2	PORin, putative mitochondrial porin (voltage-dependent anion channel)	VDAC1
YIL135C	VHS2	Viable in a Hal3 Sit4 background, regulator of septin dynamics	w/o orthologue
YIL152W	VPR1	Putative protein of unknown function	w/o orthologue
YIR016W	unass.	Putative protein of unknown function	w/o orthologue
YIR029W	DAL2	Degradation of Allantoin, allantoicase	ALLC
YJL007C		Dubious open reading frame	w/o orthologue
YJL023C	PET130	PETite colonies, protein required for respiratory growth	w/o orthologue
YJL055W	LOG1	Putative protein of unknown function	w/o orthologue
YJL062W-A	COA3	Cytochrome Oxidase Assembly, mitochondrial protein required for cytochrome c oxidase assembly	COA3
YJL095W	BCK1	Bypass of C Kinase, mAPKKK acting in the protein kinase C signaling pathway	MAP3K19
YJL096W	MRPL49	Mitochondrial Ribosomal Protein, Large subunit	MRPL21
YJL102W	MEF2	Mitochondrial Elongation Factor, mitochondrial elongation factor involved in translational elongation	GFM2
YJL116C	NCA3	Nuclear Control of ATPase, protein involved in mitochondrion organization	w/o orthologue
YJL120W	unass.	Dubious open reading frame	w/o orthologue
YJL147C	SMT1	Mitochondrial organization of gene expression, mitochondrial protein of unknown function	w/o orthologue
YJL149W	DAS1	Dst1-delta 6-Azauracil Sensitivity, putative SCF ubiquitin ligase F-box protein	w/o orthologue

YJL163C	unass.	Putative protein of unknown function	w/o orthologue
YJL192C	SOP4	Suppressor Of Pma1-7, eR-membrane protein	w/o orthologue
YJL193W	unass.	Putative protein of unknown function	w/o orthologue
YJL209W	CBP1	Cytochrome B mRNA Processing, mitochondrial protein	w/o orthologue
YJR030C	RBH2	Putative protein of unknown function	w/o orthologue
YJR031C	GEA1	Guanine nucleotide Exchange on ARF, guanine nucleotide exchange factor for ADP ribosylation factors (ARFs)	GBF1
YJR101W	RSM26	Ribosomal Small subunit of Mitochondria, mitochondrial ribosomal protein of the small subunit	w/o orthologue
YJR113C	RSM7	Ribosomal Small subunit of Mitochondria, mitochondrial ribosomal protein of the small subunit	MRPS7
YJR137C	MET5	METhionine requiring, sulfite reductase beta subunit	w/o orthologue
YJR144W	MGM101	Mitochondrial Genome Maintenance, protein with a role in mitochondrial DNA recombinational repair	w/o orthologue
YJR146W	unass.	Protein of unknown function	w/o orthologue
YKL002W	DID4	Doa4-Independent Degradation, class E Vps protein of the ESCRT-III complex	CHMP2A
YKL003C	MRP17	Mitochondrial Ribosomal Protein, mitochondrial ribosomal protein of the small subunit	MRPS6
YKL073W	LHS1	Lumenal Hsp Seventy, molecular chaperone of the endoplasmic reticulum lumen	HYOU1
YKL097C	unass.	Dubious open reading frame	w/o orthologue
YKL137W	CMC1	Cx9C Mitochondrial protein necessary for full assembly of Cytochrome c oxidase, copper-binding protein of the mitochondrial intermembrane space	CMC1
YKL169C	unass.	Dubious open reading frame	w/o orthologue
YKL170W	MRPL38	Mitochondrial Ribosomal Protein, Large subunit	w/o orthologue
YKL174C	TPO5	Transporter of POlyamines, protein involved in excretion of putrescine and spermidine	w/o orthologue
YKL187C	FAT3	FATty acid transporter 3, protein required for fatty acid uptake	w/o orthologue
YKL202W	unass.	Dubious open reading frame	w/o orthologue
YKR035W-A	DID2	Doa4-Independent Degradation, class E protein of the vacuolar protein-sorting (Vps) pathway	CHMP1A, CHMP1B

YKR046C	PET10	PETite colonies, protein of unknown function that localizes to lipid particles	w/o orthologue
YKR085C	MRPL20	Mitochondrial Ribosomal Protein, Large subunit	w/o orthologue
YKR088C	TVP38	Tlg2-Vesicle Protein, integral membrane protein	TMEM64
YKR093W	PTR2	Peptide TRansport, integral membrane peptide transporter	SLC15A1
YKR097W	PCK1	Phosphoenolpyruvate CarboxyKinase, phosphoenolpyruvate carboxykinase	w/o orthologue
YLL018C-A	COX19	Cytochrome c OXidase, protein required for cytochrome c oxidase assembly	COX19
YLL033W	IRC19	Increased Recombination Centers, putative protein of unknown function	w/o orthologue
YLL039C	UBI4	Ubiquitin, ubiquitin	UBB, UBC
YLL047W	unass.	Dubious open reading frame	w/o orthologue
YLL054C	unass.	Putative protein of unknown function with similarity to Pip2p	w/o orthologue
YLR025W	SNF7	Sucrose NonFermenting, one of four subunits of the ESCRT-III complex	CHMP4A, CHMP4B
YLR038C	COX12	Cytochrome c OXidase, subunit VIb of cytochrome c oxidase	COX6B1,3
YLR055C	SPT8	SuPpressor of Ty, subunit of the SAGA transcriptional regulatory complex	w/o orthologue
YLR056W	ERG3	ERGosterol biosynthesis, c-5 sterol desaturase	SC5D
YLR067C	PET309	PETite colonies, specific translational activator for the COX1 mRNA	w/o orthologue
YLR069C	MEF1	Mitochondrial Elongation Factor, mitochondrial elongation factor involved in translational elongation	GFM1
YLR091W	GEP5	GEnetic interactors of Prohibitins, protein of unknown function	w/o orthologue
YLR096W	KIN2	KINase, serine/threonine protein kinase involved in regulation of exocytosis	SIK3
YLR098C	CHA4	Catabolism of Hydroxy Amino acids, dNA binding transcriptional activator	w/o orthologue
YLR137W	RKM5	Ribosomal lysine (K) Methyltransferase 5, protein lysine methyltransferase	w/o orthologue
YLR139C	SLS1	Synthetic Lethal with SSM4, mitochondrial membrane protein	w/o orthologue
YLR148W	PEP3	carboxyPEPtidase Y-deficient, component of CORVET membrane tethering complex	VPS18

YLR150W	STM1	Suppressor of ToM1, protein required for optimal translation under nutrient stress	w/o orthologue
YLR151C	PCD1	Peroxisomal Coenzyme A Diphosphatase, 8-oxo-dGTP diphosphatase	NUDT7, NUDT8
YLR183C	TOS4	Target Of Sbf, putative transcription factor	w/o orthologue
YLR201C	COQ9	COenzyme Q, protein required for ubiquinone biosynthesis and respiratory growth	COQ9
YLR202C	unass.	Dubious open reading frame	w/o orthologue
YLR203C	MSS51	Mitochondrial Splicing Suppressor, specific translational activator for the mitochondrial COX1 mRNA	w/o orthologue
YLR209C	PNP1	purine nucleoside phosphorylase, purine nucleoside phosphorylase	PNP
YLR282C	unass.	Dubious open reading frame	w/o orthologue
YLR285W	NNT1	Nicotinamide N-methylTransferase, s-adenosylmethionine-dependent methyltransferase	METTL21C
YLR337C	VRP1	VeRProlin, verprolin	WIPF1, WIPF2
YLR363C	NMD4	Nonsense-Mediated mRNA Decay, protein that may be involved in nonsense-mediated mRNA decay	w/o orthologue
YLR382C	NAM2	Nuclear Accommodation of Mitochondria, mitochondrial leucyl-tRNA synthetase	LARS2
YLR396C	VPS33	Vacuolar Protein Sorting, aTP-binding protein that is a subunit of the HOPS and CORVET complexes	VPS33A
YLR429W	CRN1	CoRoNin, coronin	CORO1A
YLR431C	ATG23	AuTophaGy related, peripheral membrane protein required for autophagy and CVT	w/o orthologue
YLR439W	MRPL4	Mitochondrial Ribosomal Protein, Large subunit	MRPL47
YLR446W	unass.	Putative hexokinase	w/o orthologue
YML008C	ERG6	ERGosterol biosynthesis, delta(24)-sterol C-methyltransferase	METTL27
YML108W	unass.	Protein of unknown function	w/o orthologue
YML121W	GTR1	GTp binding protein Resemblance, cytoplasmic GTPase	RRAGA
YMR024W	MRPL3	Mitochondrial Ribosomal Protein, Large subunit	MRPL44
YMR025W	CSI1	Cop9 Signalosome Interactor, subunit of the Cop9 signalosome	w/o orthologue
YMR030W	RSF1	ReSpiration Factor, protein required for respiratory growth	w/o orthologue

YMR032W	HOF1	Homolog Of cdc Fifteen, protein that regulates actin cytoskeleton organiation	GAS7, PSTPIP1
YMR035W	IMP2	Inner Membrane Protease, catalytic subunit of mitochondrial inner membrane peptidase complex	IMMP2L
YMR040W	YET2	Yeast Endoplasmic reticulum Transmembrane protein, protein of unknown function that may interact with ribosomes	BCAP31
YMR044W	IOC4	Iswi One Complex, member of a complex (Isw1b) with Isw1p and Ioc2p	HDGF, HDGFL1
YMR064W	AEP1	ATPase ExPression, protein required for expression of the mitochondrial OLI1 gene	w/o orthologue
YMR072W	ABF2	ARS-Binding Factor, mitochondrial DNA-binding protein	HMGB1, HMGB2
YMR086W	SEG1	Stability of Eisosomes Guaranteed, component of eisosome required for proper eisosome assembly	w/o orthologue
YMR089C	YTA12	Yeast Tat-binding Analog, mitochondrial inner membrane m-AAA protease component	AFG3L2
YMR111C	EUC1	Protein of unknown function	w/o orthologue
YMR119W	ASI1	Amino acid Sensor-Independent, subunit of the nuclear inner membrane Asi ubiquitin ligase complex	w/o orthologue
YMR122C	unass.	Dubious open reading frame	w/o orthologue
YMR150C	IMP1	Inner Membrane Protease, catalytic subunit of mitochondrial inner membrane peptidase complex	IMMP1L
YMR158W	MRPS8	Mitochondrial Ribosomal Protein, Small subunit	w/o orthologue
YMR159C	ATG16	AuTophaGy related, conserved protein involved in autophagy	w/o orthologue
YMR174C	PAI3	Proteinase A Inhibitor, cytoplasmic proteinase A (Pep4p) inhibitor	w/o orthologue
YMR228W	MTF1	Mitochondrial Transcription Factor, mitochondrial RNA polymerase specificity factor	w/o orthologue
YNL068C	FKH2	ForK head Homolog, forkhead family transcription factor	FOXK1, FOXK2
YNL074C	MLF3	Multicopy suppressor of LeFlunomide sensitivity, serine-rich protein of unknown function	w/o orthologue
YNL081C	SWS2	Sick Without Securin, putative mitochondrial ribosomal protein of the small subunit	w/o orthologue
YNL093W	YPT53	Yeast Protein Two, stress-induced Rab family GTPase	RAB5A

YNL095C	unass.	Putative protein of unknown function	w/o orthologue
YNL097C	PHO23	PHOsphate metabolism, component of the Rpd3L histone deacetylase complex	ING1, ING2, ING4
YNL122C	MRP35	Putative protein of unknown function	w/o orthologue
YNL143C	unass.	Protein of unknown function	w/o orthologue
YNL177C	MRPL22	Mitochondrial Ribosomal Protein, Large subunit	MRPL22
YNL183C	NPR1	Nitrogen Permease Reactivator, protein kinase	w/o orthologue
YNL184C	unass.	Protein of unknown function	w/o orthologue
YNL190W	unass.	Hydrophilin essential in desiccation-rehydration process	w/o orthologue
YNL194C	unass.	Integral membrane protein	w/o orthologue
YNL196C	SLZ1	Sporulation-specific protein with a leucine zipper motif	w/o orthologue
YNL201C	PSY2	Platinum SensitivitY, subunit of protein phosphatase PP4 complex	PPP4R3A, PPP4R3B
YNL208W	unass.	Protein of unknown function	w/o orthologue
YNL213C	RRG9	Required for Respiratory Growth, protein of unknown function	NGRN
YNL219C	ALG9	Asparagine-Linked Glycosylation, mannosyltransferase	ALG9
YNL225C	CNM67	Chaotic Nuclear Migration, component of the spindle pole body outer plaque	w/o orthologue
YNL252C	MRPL17	Mitochondrial Ribosomal Protein, Large subunit	MRPL46
YNR036C	MRPS12	Mitochondrial Ribosomal Protein, Small subunit	MRPS12
YNR041C	COQ2	COenzyme Q, para hydroxybenzoate polyprenyl transferase	COQ2
YNR074C	AIF1	Apoptosis-Inducing Factor, mitochondrial cell death effector	AIFM2
YNR075W	COS10	Endosomal protein involved in turnover of plasma membrane protein	w/o orthologue
YOL004W	SIN3	Switch INdependent, component of both the Rpd3S and Rpd3L histone deacetylase complexes	SIN3A, SIN34
YOL007C	CSI2	Chitin Synthesis Involved, protein of unknown function	w/o orthologue
YOL009C	MDM12	Mitochondrial Distribution and Morphology, mitochondrial outer membrane protein	w/o orthologue
YOL013W-A	unass.	Putative protein of unknown function	w/o orthologue
YOL033W	MSE1	Mitochondrial aminoacyl-tRNA Synthetase, Glutamate (E)	EARS2
YOL104C	NDJ1	NonDisJunction, meiosis-specific telomere protein	w/o orthologue

YOL105C	WSC3	Cell Wall integrity and Stress response Component, sensor-transducer of the stress-activated PKC1-MPK1 signaling pathway	w/o orthologue
YOL129W	VPS68	Vacuolar Protein Sorting, vacuolar membrane protein of unknown function	TMEM50A, TMEM50B
YOR125C	CAT5	CATabolite repression, protein required for ubiquinone (Coenzyme Q) biosynthesis	COQ7
YOR175C	ALE1	Acyltransferase for Lyso-phosphatidylEthanolamine, broad-specificity lysophospholipid acyltransferase	w/o orthologue
YOR187W	TUF1	Mitochondrial translation elongation factor Tu	TUFM
YOR253W	NAT5	N-terminal AcetylTransferase, subunit of protein N-terminal acetyltransferase NatA	NAA50
YOR269W	PAC1	Perish in the Absence of Cin8p, involved in nuclear migration	PAFAH1B1
YOR277C	unass.	Dubious open reading frame	w/o orthologue
YOR285W	RDL1	RhoDanese-Like protein, thiosulfate sulfurtransferase	TSTD1
YOR298C-A	MBF1	Multiprotein Bridging Factor, transcriptional coactivator	EDF1
YOR300W	unass.	Dubious open reading frame	w/o orthologue
YOR330C	MIP1	MItochondrial DNA Polymerase, mitochondrial DNA polymerase gamma subunit	POLG
YOR344C	TYE7	Ty1-mediated Expression, serine-rich protein that contains a bHLH DNA binding motif	SREBF1, USF1
YOR346W	REV1	REVersionless, deoxycytidyl transferase	REV1
YOR360C	PDE2	PhosphoDiEsterase, high-affinity cyclic AMP phosphodiesterase	w/o orthologue
YOR364W	unass.	Dubious open reading frame	w/o orthologue
YOR378W	AMF1	AMmonium Facilitator, low affinity NH4+ transporter	w/o orthologue
YPL005W	AEP3	ATPase ExPression, peripheral mitochondrial inner membrane protein	w/o orthologue
YPL013C	MRPS16	Mitochondrial Ribosomal Protein, Small subunit	MRPS16
YPL065W	VPS28	Vacuolar Protein Sorting, component of the ESCRT-I complex	VPS28
YPL078C	ATP4	ATP synthase, subunit b of the stator stalk of mitochondrial F1F0 ATP synthase	ATP5PB
YPL104W	MSD1	Mitochondrial aminoacyl-tRNA Synthetase, Aspartate (D)	DARS2

YPL132W	COX11	Cytochrome c OXidase, protein required for delivery of copper to Cox1p	COX11
YPL189C-A	COA2	Cytochrome Oxidase Assembly, cytochrome oxidase assembly factor	w/o orthologue
YPL216W	unass.	Putative protein of unknown function	w/o orthologue
YPL247C	unass.	Putative protein of unknown function	DCAF7
YPR023C	EAF3	Esa1p-Associated Factor, component of the Rpd3S histone deacetylase complex	MORF4L1, MORF4L2
YPR036W	VMA13	Subunit H of the V1 peripheral membrane domain of V-ATPase	ATP6V1H
YPR047W	MSF1	Mitochondrial aminoacyl-tRNA Synthetase, Phenylalanine (F)	FARS2
YPR060C	ARO7	AROmatic amino acid requiring, chorismate mutase	w/o orthologue
YPR100W	MRPL51	Mitochondrial Ribosomal Protein, Large subunit	MRPL43
YPR155C	NCA2	Nuclear Control of ATPase, protein that regulates expression of Fo-F1 ATP synthase subunits	w/o orthologue
YPR172W	unass.	Putative pyridoxal 5'-phosphate synthase	w/o orthologue

unass. = unassigned
w/o orthologue = without mammalian orthologue

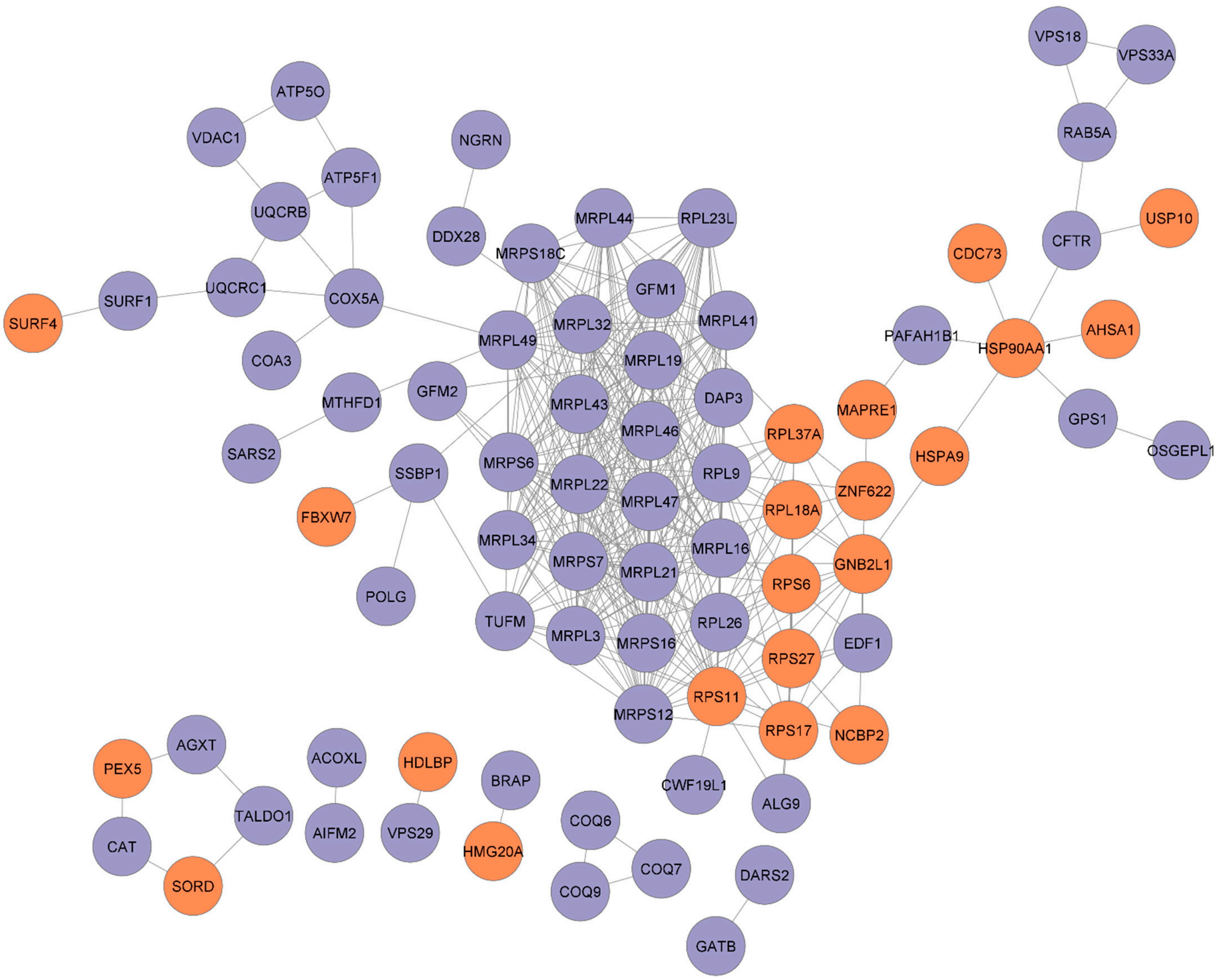

Figure S6. Amyloid toxicity protective genes can be seen in blue and activator genes can be seen in salmon. The layout of the network is organic.

Table S3. Network analysis results of amyloid toxicity activator and protective Genes. Top 10 best results for average shortest path length, degree, and betweenness centrality

Average Shortest Path Length	Value	Property	Degree	Value	Property	Betweenness Centrality	Value	Property
VPS18	6.63	protective	*MRPS7*	30	protective	*GNB2L1*	0.33	enhancer
VPS33A	6.63	protective	*MRPS12*	29	protective	*MRPL49*	0.29	protective
SURF4	6.05	enhancer	*RPL9*	28	protective	*HSP90AA1*	0.25	enhancer
OSGEPL1	5.82	protective	*RPS11*	28	enhancer	*HSPA9*	0.25	enhancer
USP10	5.72	enhancer	*MRPL22*	26	protective	*COX5A*	0.22	protective
RAB5A	5.66	protective	*MRPL3*	26	protective	*AGXT*	0.17	protective
ATP5O	5.09	protective	*GFM1*	25	protective	*TALDO1*	0.17	protective
SURF1	5.06	protective	*MRPL19*	25	protective	*PEX5*	0.17	enhancer
VDAC1	5.05	protective	*MRPL21*	25	protective	*CAT*	0.17	protective
AHSA1	4.86	enhancer	*MRPS16*	25	protective	*SORD*	0.17	enhancer

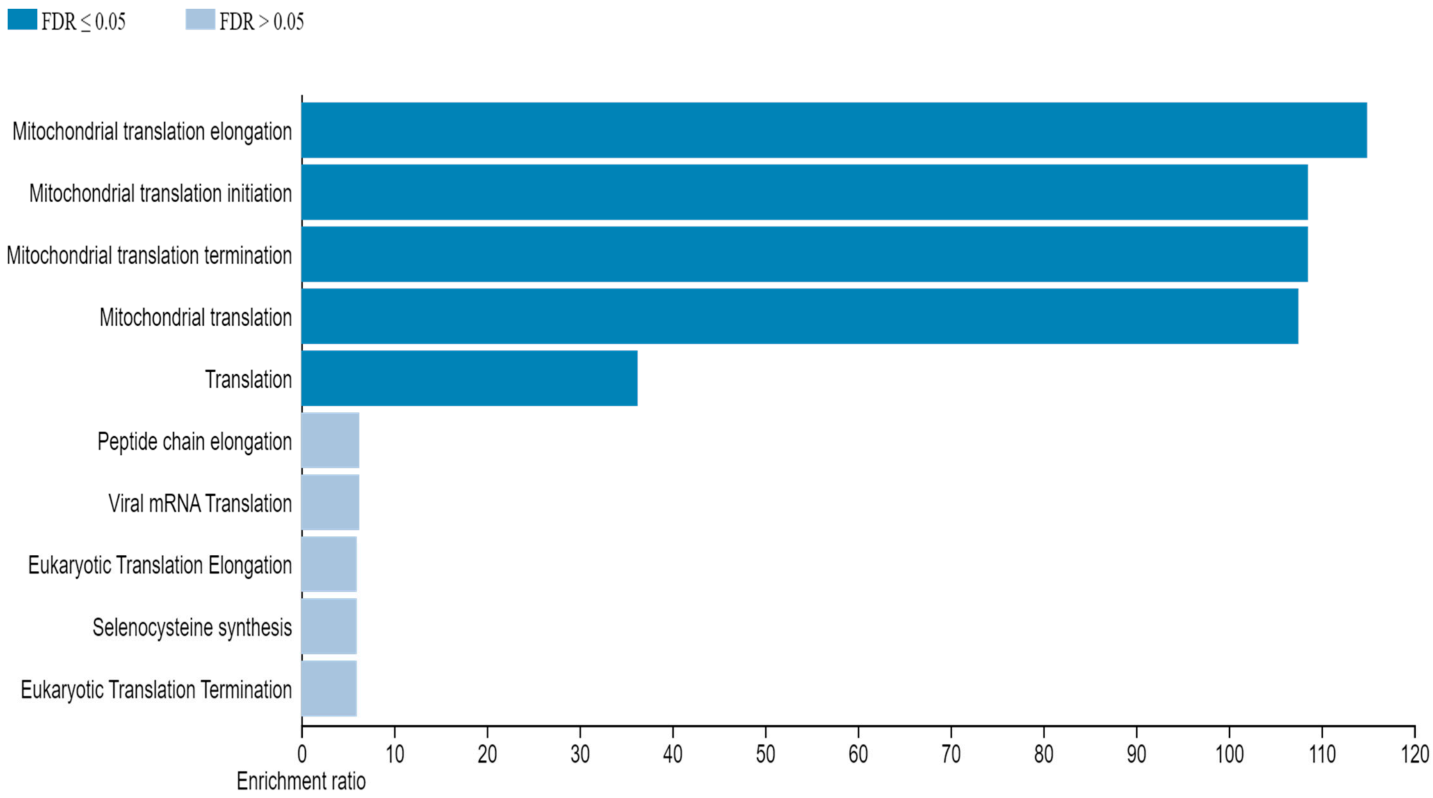

Figure S7. The chart demonstrates the Reactome pathways enriched in the genes which form the highest scoring clusters according MCODE (Figure 4 - yellow highlighted genes).

Table S4. Text-mining of the amyloid toxicity enhancer yeast genes

	GO term name	Number of genes	Total gene number	Adj. P-value
4	SCF-dependent proteasomal ubiquitin-dependent protein catabolic process	3	19	3,40E+06
6	positive regulation of ubiquitin-dependent protein catabolic process	2	10	0.00123567025803
6	protein ubiquitination	3	135	0.00448291121121
9	negative regulation of cellular response to tunicamycin	1	1	0.00551631746404
9	positive regulation of hepatic stellate cell activation	1	1	0.00551631746404
4	negative regulation of store-operated calcium channel activity	1	1	0.00551631746404
9	positive regulation of neurofibrillary tangle assembly	1	1	0.00551631746404
	microglial cell proliferation	1	1	0.00551631746404
7	negative regulation of response to endoplasmic reticulum stress	1	1	0.00551631746404
9	positive regulation of protein ubiquitination	2	47	0.00551631746404
9	negative regulation of cellular response to thapsigargin	1	1	0.00551631746404
4	regulation of neuronal signal transduction	1	1	0.00551631746404
3	negative regulation of SREBP signaling pathway	1	1	0.00551631746404
4	negative regulation of hepatocyte proliferation	1	2	0.00754574460057
6	negative regulation of triglyceride biosynthetic process	1	2	0.00754574460057
2	regulation of amyloid-beta clearance	1	2	0.00754574460057
7	regulation of oxidative stress-induced intrinsic apoptotic signaling pathway	1	2	0.00754574460057
7	hepatocyte proliferation	1	2	0.00754574460057
7	positive regulation of oxidative stress-induced neuron intrinsic apoptotic signaling pathway	1	2	0.00754574460057
3	central nervous system myelin maintenance	1	3	0.00859884385703
3	protein import	1	3	0.00859884385703
0	negative regulation of osteoclast development	1	3	0.00859884385703
7	microglial cell activation	1	3	0.00859884385703
0	negative regulation of amyloid fibril formation	1	3	0.00859884385703
3	response to misfolded protein	1	3	0.00859884385703

Table S5. Text-mining of the amyloid toxicity protective yeast genes

GO ID	GO term name	Number of genes	Total gene number	Adj. P-value
0051403	stress-activated MAPK cascade	3	10	9,21E+04
0070849	response to epidermal growth factor	2	3	3,00E+06
1903351	cellular response to dopamine	2	7	0.000139883410432
0018105	peptidyl-serine phosphorylation	3	81	0.000161554822097
1904355	positive regulation of telomere capping	2	16	0.000478494983168
0071276	cellular response to cadmium ion	2	21	0.000696910906528
0016310	phosphorylation	2	25	0.000745924397294
0034614	cellular response to reactive oxygen species	2	24	0.000745924397294
0034198	cellular response to amino acid starvation	2	32	0.000895307729786
0032212	positive regulation of telomere maintenance via telomerase	2	30	0.000895307729786
0051973	positive regulation of telomerase activity	2	31	0.000895307729786
0018107	peptidyl-threonine phosphorylation	2	40	0.00128796663344
0072709	cellular response to sorbitol	1	1	0.00278982126847
0072740	cellular response to anisomycin	1	1	0.00278982126847
0035408	histone H3-T6 phosphorylation	1	2	0.00520633243682
0031281	positive regulation of cyclase activity	1	3	0.00688896906305
0050861	positive regulation of B cell receptor signaling pathway	1	3	0.00688896906305
0010829	negative regulation of glucose transmembrane transport	1	4	0.00821631410542
0038127	ERBB signaling pathway	1	4	0.00821631410542
0033129	positive regulation of histone phosphorylation	1	5	0.00886761254667
1903936	cellular response to sodium arsenite	1	5	0.00886761254667
0048709	oligodendrocyte differentiation	1	5	0.00886761254667
0006975	DNA damage induced protein phosphorylation	1	6	0.0093618002335
0030949	positive regulation of vascular endothelial growth factor receptor signaling pathway	1	6	0.0093618002335
0035094	response to nicotine	1	6	0.0093618002335
0042113	B cell activation	1	7	0.00974937732528
0006970	response to osmotic stress	1	7	0.00974937732528
0051966	regulation of synaptic transmission, glutamatergic	1	7	0.00974937732528

Table S6. Primer sequences used for experiments

**qPCR primer**	**Sequence (5'-3')**
hSURF4-Forward	CTATTGTCCAGAACATCGTG
hSURF4-Reverse	CCAGAAGGCGTTGAAATAT
hGAPDH-Forward	GGAGTCCACTGGCGTCTTC
hGAPDH-Reverse	TGGCTCCCCCCTGCAAATG
hHPRT-Forward	TGACACTGGCAAAACAATGCA
hHPRT-Reverse	GGTCCTTTTCACCAGCAAGCT

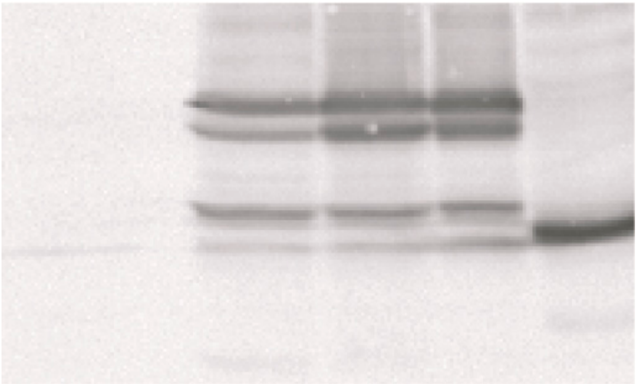

Figure S8. Uncropped western blot images showed in Figure 2B.
